# A novel fucoxanthin enriched seaweed gummy: Physicochemical qualities and protective effect on UVB-induced retinal müller cells

**DOI:** 10.1016/j.fochx.2024.101648

**Published:** 2024-07-14

**Authors:** Yu Liu, Yixin Shi, Yuting Wang, Zhipeng Wang, Yuze Wang, Yujing Lu, Hang Qi

**Affiliations:** National Engineering Research Center for Seafood, State Key Laboratory of Marine Food Processing and Safety Control, Collaborative Innovation Center of Provincial and Ministerial Co-construction for Seafood Deep Processing, Liaoning Province Collaborative Innovation Center for Marine Food Deep Processing, Dalian Technology Innovation Center for Chinese Pre-made Food, College of Food Science and Technology, Dalian Polytechnic University, Dalian 116034, China

**Keywords:** Fucoxanthin, Seaweed gummies, Physicochemical quality, Photoprotection

## Abstract

Retinal disease has become the major cause of visual impairment and vision loss worldwide. Carotenoids, which have the potential antioxidant and eye-care activities, have been widely used in functional foods. Our previous study showed that fucoxanthin could exert photoprotective activity in UVB-induced retinal müller cells (RMCs). To extend the application of fucoxanthin in food industry, fucoxanthin, *Undaria pinnatifida* pulp (UPP), carrageenan, and other ingredients were mixed to prepare seaweed-flavoured photoprotective gummies in this study. The structural and functional properties of the gummies were then evaluated by physicochemical test and cell experiments. As a result, fucoxanthin enriched gummies presented favourable structural properties and flavour. The hydroxyl groups in fucoxanthin and κ-carrageenan are bonded through hydrogen bonds, forming the spatial network structure inside the gummies, enhancing its elasticity. The gummies showed significant antioxidant effect and alleviated the UVB oxidation damage in RMCs. Moreover, the main ingredients carrageenan and UPP improved the stability of fucoxanthin during *in vitro* digestion. The results enhance the application of fucoxanthin in functional food with photoprotective activity.

## Highlights

• A novel fucoxanthin enriched seaweed gummies were prepared.
• The gummies presented favourable structural properties and flavour.
• The gummies alleviated the UVB oxidation damage in retinal müller cells.
• The main ingredients improved the stability of fucoxanthin during *in vitro* digestion.

## Graphical abstract

**Figure:**
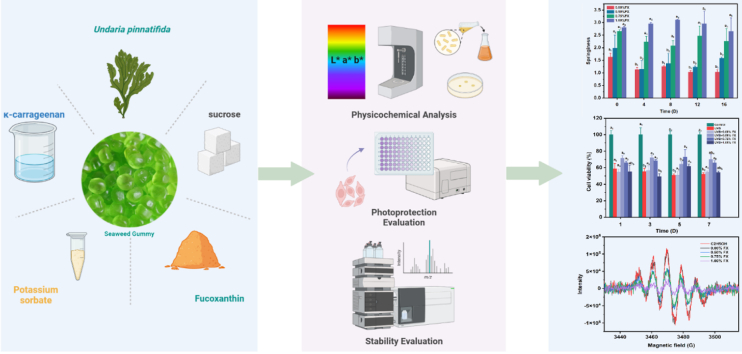

## 1 Introduction

Retinal damage has become a common health problem that affects people of all ages (Hiramoto et al., 2020). With the gradually overuse of mobile phones and computers, the long-term illumination usually causes the injury in ocular tissues. Generally, the photodamage in retina caused by the stimulation of blue light and ultraviolet (UV) exposure is associated with oxidative stress that results in free radical accumulation and cell apoptosis (Hsu et al., 2022). Studies have found that ultraviolet B (UVB) mainly cause severe damage to retina, resulting in a high level of reactive oxygen species (ROS) in the retina (Zhao et al., 2014). Thus, the importance of retinal health care has become increasingly clear.

Some natural compounds can ameliorate the oxidation damage and have the potential antioxidative effects, which made it possible to prevent the photodamage in retina. Recently, interest in employing bioactive compounds from natural sources to produce eye-care functional foods is considerably enhanced by consumer preference for natural ingredients and concerns about the toxic effects of drugs(Drosou et al., 2022). Those functional foods that relieve visual fatigue mainly include vitamins, anthocyanins, and carotenoids(Drosou et al., 2022; Wang et al., 2016). Since carotenoids have the functions of improving vision, they have been widely used as food additives in food, beverages, and even infant foods. (Mitra et al., 2021) reported that the intake of carotenoid by food or nutritional supplements is beneficial for vision problems such as age-related macular degeneration and diabetic retinopathy. Fucoxanthin is the most abundant carotenoid in algae, which is also potent antioxidant, anti-inflammation and photoprotection agents (Yusof et al., 2022). Fucoxanthin extracts from *Laminaria japonica* have cleared intracellular ROS and protected retina against photoinduced damage both *in vitro* and *in vivo* (Liu et al., 2016). Moreover, (Chiang et al., 2020) studied the therapeutic effects of fucoxanthin on retinal diseases. In diabetic retinal cell models, fucoxanthin improved the integrity of the blood/retinal barrier. Thus, these functional potentials of fucoxanthin make it ideal candidatures for functional food and nutraceutical formulations which may help mitigate the risks of retinal diseases.

Our previous study showed that fucoxanthin could exert photoprotective effect in retinal cells, reduced UVB-induced oxidative damage in retinal müller cells (RMCs) (Shi et al., 2022). However, the application potential of fucoxanthin as a functional food remains to be explored. And the daily intake may vary significantly due to the various taste preferences of consumers. According to the study by (DeMars & Ziegler, 2001), one such food product that is usually desired by any age group is gummy which is popular for the favourable elasticity and chewy taste among consumers. Nutraceutical gummies are novel formulations ideal for kids and adults and are known to be functional gummies (Dille et al., 2018).

In this study, a novel fucoxanthin enriched seaweed gummy was prepared as a photoprotective product. The texture profile of the gummy was evaluated to demonstrate the effects of fucoxanthin addition at different concentrations. More specifically, other physicochemical properties of the gummies, including colour changes, water status, sensory evaluation, and bacterial count were analysed. The physicochemical changes of gummies were determined in the accelerated storage process. Next, the antioxidative and photoprotective activity of the seaweed gummy were explored based on the UVB-induced RMCs model. Finally, the simulated *in vitro* digestion was performed to evaluate the bioavailability of fucoxanthin in the gummy product. The fucoxanthin enriched seaweed gummy prepared in this study provides various health benefits and is also desirable for consumption. In addition, the results may aid in the development of functional foods with fucoxanthin as the main component.

## 2 Materials and method

### 2.1 Chemicals reagents

Sucrose, κ-carrageenan (food grade), and potassium sorbate were purchased from Henan Wanbang Industrial Co., Ltd. (Henan, China). Salted UP was purchased from Dalian Mariculture Group Co., Ltd. (Dalian, China) and stored at 4 °C. FX (≥ 98% purity) was purchased from Chengdu Desite Biotechnology Co., Ltd. (Chengdu, China). fetal bovine serum (FBS) was obtained from Shenggong Bioengineering Co., Ltd. (Shanghai, China). RMCs were provided by the Cell Bank of TongPai Biological Technology Co., Ltd. (Shanghai, China) and were cultured in Dulbecco's minimal essential medium (DMEM) with 10% FBS and 100 U/mL penicillin and streptomycin. DMEM and 0.25% trypsin-EDTA were obtained from Gibco (Thermo Fisher, Fair Lawn, NJ, USA). Penicillin and streptomycin were obtained from HyClone (Logan, UT). 3-(4,5-Dimethylthiazol-2-yl)-2,5-diphenyltetrazolium bromide (MTT), phosphate-buffered saline (PBS), and dimethyl sulfoxide (DMSO) were sourced from Solarbio Technology Co., Ltd. (Beijing, China). Sodium hydroxide, zinc acetate, and glacial acetic acid were purchased from Tianjin Damao Chemical Reagent Co., Ltd. (Tianjin, China). Yeast extract, and sodium alginate were obtained from Shanghai Macklin Biochemical Technology Co., Ltd. (Shanghai, China). Tryptone, glucose, agar, and crystal violet neutral red bile salt glucose agar were from Qingdao Haibo Biotechnology Co., Ltd. (Qingdao, China). Hydrochloric acid was purchased from Chengdu Chron chemical Co., Ltd. (Chengdu, China). All other chemicals were used without further purification.

### 2.2 Preparation of UP pulp (UPP)

The salted UP was soaked in deionized water for 15 min and then washed for 3 times to desalt. The intact verdant green UP without stains on the leaves was then selected to prepare the pulp. UP was then weighed and mixed with deionized water at a ratio of 2:1 (*w*/*v*) before grinded into UPP.

### 2.3 Preparation of fucoxanthin enriched seaweed gummies

According to GB 17399–2016 standards for candy, SB/T10021–2017 standards for gelatinized candy, and GB2760–2014 hygienic standards for uses of food additives, the fucoxanthin enriched seaweed gummies was made as follows: 30% UPS, 15% sucrose, 2% κ-carrageenan, 0.5%–1% fucoxanthin, and 0.05% potassium sorbate. Seaweed gummies without fucoxanthin was established to investigate the effect of different concentrations of fucoxanthin on the properties of gummies. Briefly, κ-carrageenan was heated to dissolve in a water bath at 80 °C. UPS was mixed with sucrose, fucoxanthin (0%, 0.5%, 0.75%, 1%), and potassium sorbate. The mixture was then added to the dissolved κ-carrageenan solution when the cooling temperature was below 60 °C. A specific mold was used to solidified the gummies.

### 2.4 Texture profile analysis (TPA)

TPA analysis was used to determine the texture profile of fucoxanthin enriched seaweed gummies. Before measurement, the instrument was adjusted to the optimal parameters, and the sample was placed on the platform. TPA analysis was performed at room temperature, with the probe model of P/50. The parameters were set with a pre-test speed of 2 mm/s, test speed of 1 mm/s, post-test speed of 2 mm/s, strain of 20%, compression time of 5 s, and trigger force of 5 g. The hardness (N), springiness, and adhesiveness were measured to evaluate the quality of seaweed gummies(Herrero et al., 2008).

### 2.5 Colour measurement of fucoxanthin enriched seaweed gummies

The gummies were cut into samples with a thickness of 5 mm, and the changes in brightness (L*), redness to greenness (a*), and yellowness to blueness (b*) values of different groups of gummies were measured by using a colorimeter.

### 2.6 Low field-nuclear magnetic resonance (LF-NMR) analysis

The water status of the seaweed gummies was measured by using a LF-NMR analyser according to the method of (L. Li et al., 2019), with slight modifications. The key parameters were set as follows: echo time (TE) of 0.5 ms, waiting time (TW) of 4000 ms, number of scan (NS) of 8, and number of echo (NECH) of 8000.

### 2.7 Sensory evaluation

The sensory evaluation of seaweed gummies was conducted based on the method referenced by (Chen et al., 2021). In this study, 20 volunteers (10 males and 10 females) aged between 21 and 30 were selected as professional trained evaluators. All the testers were in normal weight range (BMI < 30 kg/m^2^), and had no functional impairments, allergies or diseases that could affect sensory abilities and judgment. Other exclusion criteria included food preferences, dental braces, diabetes, or pregnancy. This study was exempted from the need for formal ethical permission. Before the experiment, we ensured that the internal participants were safe, voluntary, and knowledgeable about the experiment. Moreover, all data presented in this study were collected with the participants' informed consent, and the participants were informed how the data would be used.

The sensory evaluation criteria of seaweed gummies include colour, texture, flavour, and taste. Each criterion accounts for 25 points respectively, and the overall score of the gummy was on a 100-point scale. All samples were evaluated blindly in triplicate. The test was performed at room temperature.

### 2.8 Antioxidant analysis of gummies

The DPPH scavenging rate of seaweed gummy extract was detected by electron spin resonance spectroscopy (ESR) with the method of (Qi, Dong, et al., 2016). Briefly, the extract of different fucoxanthin enriched gummies was mixed with DPPH solution and kept in dark for 30 min. Thereafter, the samples were centrifuged at 2000 ×*g* for 10 min, and the supernatant was kept for further detection. The ESR parameters were set according to our previous study (Qi et al., 2016).

### 2.9 Cell viability assay

The seaweed gummies were dissolved in DMSO and filtered through a 0.22 μm organic phase ultrafiltration membrane. The solution was then diluted with cell culture medium and cultured with RMCs for 24 h. Cell treatment method was performed as described previously (Shi et al., 2022). Briefly, the RMCs were cultured in DMEM supplemented with 10% FBS and penicillin/streptomycin (100 U/mL) in a 5% CO_2_ humidified atmosphere at 37 °C, as per the manufacturer's instructions. Cell viability level was evaluated by MTT assay.

### 2.10 Determination of total bacterial count (TBC) and total coliform count (TC)

The seaweed gummy samples were stored at 4 °C, and the TBC and TC were both measured on day 1 and day 7. The plate count method was applied to measure TBC and TC according to Chinese national standard GB4789.1–2016 food microbiology testing general provisions, GB4789.2–2016 food microbiology testing total bacterial count and GB 4789.3–2016 food microbiology testing total coliform count. All the tests were performed in triplicate.

### 2.11 Preparation for accelerated storage studies of fucoxanthin enriched seaweed gummies

Accelerated storage testing is commonly used to investigate the stability of products under the higher temperature and humidity conditions in long-term storage(He et al., 2021). The stability of the gummies was tested according to the method of (Agregán et al., 2017), with slight modifications. A constant temperature and humidity incubator was used to ensure the experimental condition, which were set at 37 °C and 60% humidity. Samples were taken after 0, 4, 8, 12, and 16 days to evaluate the changes in the fucoxanthin content, texture, colour, and antioxidant effect. All the analyses had at least three replicates (*n* = 3).

#### 2.11.1 Determination of fucoxanthin

To quantify fucoxanthin, 3 g of each seaweed gummy sample was dissolved in 1 mL of methanol. The solution was centrifuged at 6200 *g* for 5 min, and the supernatant was filtered through a 0.22 μm organic phase ultrafiltration membrane. The fucoxanthin content in the samples was detected using high-performance liquid chromatography (HPLC) according to the previous method (Sui et al., 2021).

#### 2.11.2 TPA and colour measurement

The texture and colour of the seaweed gummies during accelerated storage were determined every 4 days.

#### 2.11.3 Antioxidant effect

The antioxidant effect of the gummies during accelerated storage was evaluated by DPPH radical scavenging rate (Qi, Dong, et al., 2016).

### 2.12 Effect of the gummy ingredients on the stability of fucoxanthin

Alginates and κ-carrageenan used in the seaweed gummies is a polysaccharide that is considered as a carrier for embedding bioactive compounds. Therefore, fucoxanthin was mixed with sodium alginate and κ-carrageenan for further analysis. The experimental groups were set as follows: FX, fucoxanthin; FS, fucoxanthin and sodium alginate; FC, fucoxanthin and κ-carrageenan; FSC, fucoxanthin, sodium alginate and κ-carrageenan.

#### 2.12.1 Fucoxanthin content measurement after *in vitro* digestion

The four groups of gummy samples were subjected to *in vitro* digestion, according to the method reported by (C. Wang et al., 2021), with slight modification. All the digested samples were store the at −20 °C for lyophilization. HPLC was used to determine the concentration of fucoxanthin after the simulated *in vitro* digestion.

#### 2.12.2 Effect of different ingredients on the *in vitro* digestion products of fucoxanthin

The lyophilizates were dissolved in methanol to prepare the sample solution with a concentration of 1 mg/mL. The solution was then sonicated for 30 s, with the ultrasonic power at 70 W in ice-bath. Thereafter, all samples were centrifuged at 12,000 rpm for 5 min, and the supernatant was collected and filtrated. Before UPLC-QE-MS/MS analysis, the solution was diluted to a final concentration of 0.1 mg/mL. UPLC-QE-MS/MS parameters was set as described previously (Zhou et al., 2021).

### 2.13 Statistical analysis

All the tests were performed in triplicate, and the experimental data were expressed as the mean ± standard deviation (SD). SPSS 19.0 (SPSS Inc., Chicago, IL, USA) statistical analysis was used to perform one-way analysis of variance on the data obtained from each group. *P* < 0.05 was considered statistically significant.

## 3 Results and discussion

### 3.1 Effects of different concentrations of Fucoxanthin on texture and colour of gummies

κ-carrageenan was used as the main ingredients of seaweed gummy, which give gummy the properties of gel. It is a negatively charged sulfated polysaccharide with strong gelation capability and shows heightened sensitivity to cations, especially K. κ-carrageenan and potassium sorbate cross-link through electrostatic interactions, which promotes the aggregation of carrageenan helical structure and stabilizes the gel network, which is conducive to the structural stability of seaweed gummy (Wang et al., 2024). Sodium alginate has a high content of carboxyl groups and carrageenan has a high content of hydroxyl groups and at a certain proportion of compound sodium alginate and carrageenan, a high number of hydrogen bonds between the two compounds are formed, making a crosslinking network through hydrogen bonding and ionic interaction (F. Yu et al., 2019). This network maintains a relatively stable form and makes the sodium alginate and carrageenan intermolecular spacing decrease, so that space network structure becomes increasingly compact. Thus, it is beneficial for the stabilization of the structure of the seaweed gummy. Sucrose, a sweetener commonly used in desserts and confectionery, can improve the elasticity and hardness of carrageenan-based candies. It has been shown that sucrose addition to κ-carrageenan increases the gel temperature and melting temperature of carrageenan, resulting in a stronger gel network (Yang et al., 2018). The molecular interaction of sucrose with carrageenan promotes the formation of denser and thicker fiber helical connections to facilitate the formation of κ-carrageenan gels. The rheological properties were crucial in preparation of seaweed gummy and shape retention capability after preparation. In most food applications, carrageenan is often mixed with other polysaccharides and/or proteins to tune and control the rheological properties. The study shown that hydrogen bonding was identified as the major mechanism of interactions among the components of these ink gels (Qiu et al., 2024). In this study, sodium alginate and carrageenan form a compact and homogeneous gel network through hydrogen bonding, which facilitates the support properties of the shape of fondant after extrusion through the mold. The rheological properties were crucial in preparation of seaweed gummy and shape retention capability after preparation. The texture properties of seaweed gummies with different concentrations of fucoxanthin were evaluated by hardness, springiness, and chewiness. As shown in **Table S1**, the hardness and chewiness of the fucoxanthin enriched gummies were similar with the control group, indicating that the addition of fucoxanthin had no effect on the texture properties of the gummies. Likewise, the springiness of the gummies increased slightly with the increase of fucoxanthin concentration, but had no significance (*P* > 0.05).

The colour of the seaweed gummies was analysed by using a colorimeter, of which L* represents lightness, a* represents red-green values, and b* represents yellow-blue values. In **Table S2**, the addition of fucoxanthin had no significant effect on L* and b*, whereas a* increased slightly with a concentration-dependent manner. Therefore, the reddish-brown colour of fucoxanthin may distributed the colour of seaweed gummies, especially on the greenness of gummies. Notably, a* represented a negative value in all gummy samples, indicating that the gummies with low concentrations of fucoxanthin had a similar colour compared with the control group (Atambayeva et al., 2023).

### 3.2 Effects of different concentrations of Fucoxanthin on water migration in seaweed gummies

The transverse relaxation time T_2_ indicates the degree of freedom of hydrogen protons and the strength of the binding force in the sample. The longer T_2_ indicates higher water activity and greater fluidity. Integrating the relaxation peak yields the corresponding relaxation peak area (Jiang et al., 2021). **Fig. S1** shows the fitted T_2_ relaxation spectra of the gummies. Compared with the control group, the T_21_ value of fucoxanthin enriched gummies were in the range between 1.03 ± 1.38 ms and 13.48 ± 7.72 ms, suggesting that fucoxanthin caused water to migrate continuously towards immobile state. T_21_ is the shortest relaxation time component, which is defined as bound water, followed by T_22_, which is immobile water that has intermediate degrees of freedom between bound and free water and is prone to transformation. T_23_ is the longest relaxation time that reflects free water. (Younas et al., 2021). The T_23_ values of the three fucoxanthin enriched gummies were 163.48 ± 3.86, 159.59 ± 2.35, and 158.83 ± 8.72, respectively, indicating that the addition of fucoxanthin reduced the content of free water.

In addition, the changes in the proportion of LF-NMR peak area also reflects the distribution state of various water components and water migration (Atambayeva et al., 2023). It can be seen from **Fig. S1** that the proportion of A_23_ in the control group was relatively high, while the proportion of A_23_ in the fucoxanthin enriched gummies were significantly reduced in a concentration-dependent manner. This may be due to the strong antioxidant properties of fucoxanthin, which prevented the gel structure of the gummies from being oxidized by air, preventing more free water molecules from being exposed and exerting better water-holding properties (Li, Jin, et al., 2023).

### 3.3 Sensory evaluation

The sensory quality of food reflects the commercial value and influences consumer behaviour (Etaio et al., 2010). According to the study by (Ghazal et al., 2021), the attributes that have the greatest impact on consumer preference are the healthiness of the food, followed by taste, flavour, and colour. As shown in **Table S3**, the flavour and texture scores of the four groups of gummies were similar, indicating a better palatability and high flavour acceptance of the products. The indicators that mainly affected the overall scores of the four types of gummies were colour and appearance. As the concentration of fucoxanthin increased, the sensory scores for gummies colour and appearance decreased. The reason could be that the high concentration of fucoxanthin caused the darker colour of the gummies, which affected the overall sensory level (Boutheina et al., 2023). Overall, seaweed gummies with 0.5% fucoxanthin had the best shape and flavour scores, as well as the highest overall sensory score.

### 3.4 Effects of different concentrations of fucoxanthin on the antioxidant activity of gummies

**Fig. 1:**
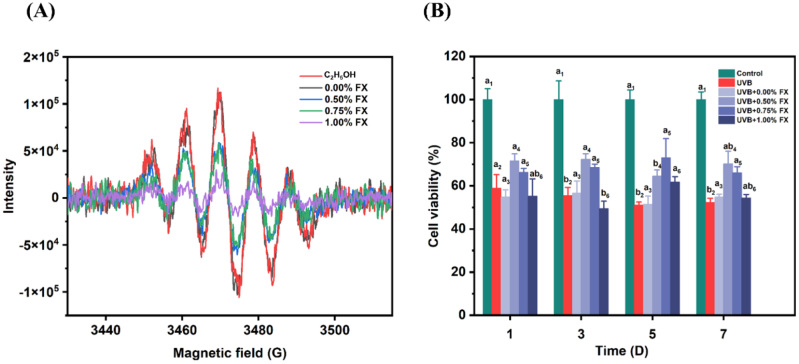
Antioxidant activity of fucoxanthin enriched seaweed gummies. (A) DPPH scavenging rate of different concentrations fucoxanthin seaweed gummies. (B) Effects of fucoxanthin seaweed gummies on cell viability. Different letters with the same subscript number indicate significant difference (*P* < 0.05).

DPPH free radical scavenging rate of the fucoxanthin-enriched seaweed gummies was performed to evaluate the antioxidant ability. Ethanol was used as a solvent to extract the effective components from the gummy samples. As illustrated in Fig. 1A, the results of ethanol group showed the similar free radical scavenging rate compared with 0% fucoxanthin group, indicating that ethanol had no effect on the results. The DPPH free radical signal peak gradually reduced with the increase of fucoxanthin concentration. Therefore, the addition of fucoxanthin in gummies exerted a significant scavenging rate of DPPH. The results were similar with the study of (Boutheina et al., 2023). The DPPH radical scavenging rates of the gummies with 0.5%, 0.75%, and 1% fucoxanthin were 51.09 ± 0.42%, 61.03 ± 2.11%, and 69.84 ± 1.60%, respectively. It can be concluded that fucoxanthin maintained a stability after being added to gummy samples and revealed significant antioxidant activity.

### 3.5 Photoprotective effect of seaweed gummies on UVB-induced RMCs

The photoprotective effect of fucoxanthin gummies on cell viability was evaluated in UVB-induced RMCs based on the previous study (Shi et al., 2022). As illustrated in Fig. 1B, UVB irradiation contributed to the decrease of cell viability, whereas the treatment of gummy extracts recovered the cell viability. Notably, the gummies containing 0.5% fucoxanthin had the best effect, with a cell viability of 71.65 ± 3.18% at day 1. With the increase of fucoxanthin concentration, the survival rate of RMCs showed a decreasing trend, indicating that the high concentrations of fucoxanthin was counterproductive to exert cytoprotective effects (Chiang et al., 2020). The results in our previous study revealed that the optimal concentration of fucoxanthin in UVB-induced RMCs was 20 μg/mL, which also explained that high concentration fucoxanthin treatment showed the inhibitory effect on cell viability.

With the extension of storage time, the effect of gummies on cell viability was slightly decreased. On the storage time of day 7, the cell viability of the groups treated with 0.5%, 0.75%, and 1% gummy extract were 70.30 ± 5.77%, 66.03 ± 2.83%, and 54.40 ± 1.64%, respectively. The results confirmed the photoprotective effect of fucoxanthin enriched gummies on RMCs, which could reduce the oxidative stress of UVB irradiation in RMCs.

### 3.6 TBC and TC evaluation

**Fig. 2:**
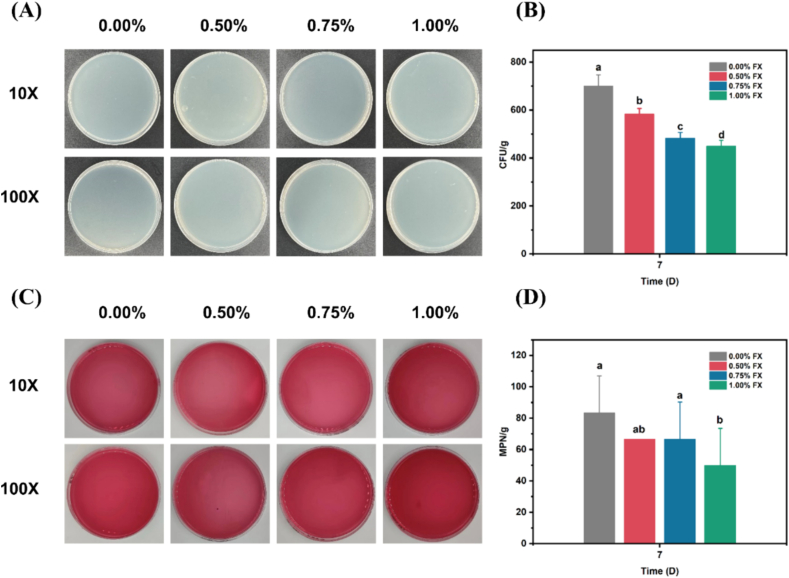
CFU and MPN of fucoxanthin seaweed eye-care gummies. (A) Appearance of CFU. (B) Quantitative analysis of CFU. (C) Appearance of MPN; (D) Quantitative analysis of MPN. Different letters indicate significant difference (*P* < 0.05).

Starting with a good quality of TBC and TC is of prime importance to guaranty the safety of product (Legan et al., 2001). According to the national standards, TBC and TC of seaweed gummies are limited to 10^5^ and 10^2^, respectively. As shown in Fig. 2A and B, TBC number of the four gummy samples were 700.00 ± 47.14 CFU/g, 583.33 ± 23.57 CFU/g, 483.33 ± 23.57 CFU/g and 450.00 ± 23.57 CFU/g, respectively. As expected, the TBC number did not exceed the detection limit after 7 days of storage. Such value confirms the good quality of the seaweed gummies.

In Fig. 2C and D, TC number of the four gummy samples were 83.33 ± 23.57 MPN/g, 66.67 ± 0.00 MPN/g, 66.67 ± 23.57 MPN/g, and 50.00 ± 23.57 MPN/g, respectively. With the increase of fucoxanthin concentration, the TBC and TC number in the gummies showed a decreasing trend, indicating that fucoxanthin exerted antibacterial effect, thus inhibiting harmful microorganisms in the gummies (Burgos-Díaz et al., 2022).

### 3.7 Changes in quality indicators of seaweed gummies during accelerated storage

#### 3.7.1 Fucoxanthin content in gummies

**Fig. 3:**
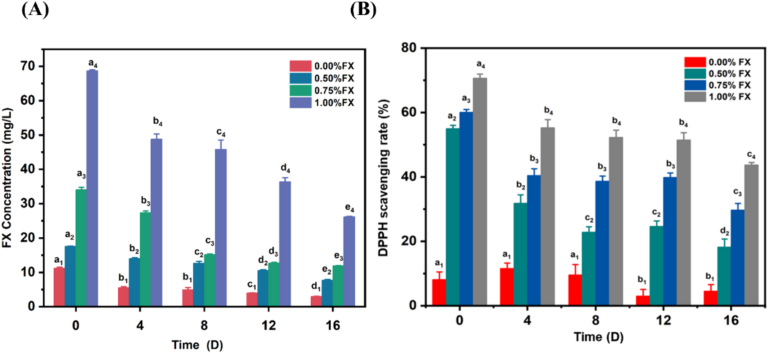
Evaluation of fucoxanthin enriched seaweed gummies during accelerated storage. (A) Changes of fucoxanthin in gummies during accelerated storage. (B) Changes of antioxidant level of fucoxanthin seaweed eye-care gummies during accelerated storage. Different letters with the same subscript number indicate significant difference (*P* < 0.05).

The changes in fucoxanthin content of gummies during accelerated storage were determined by using high-performance liquid chromatography. Fucoxanthin is susceptible to degradation under the high temperature and humidity conditions during accelerated storage (Nurcahyanti et al., 2023). As shown in Fig. 3A, the concentration of fucoxanthin decreased with the storage time, and the data revealed significant differences (*P* < 0.05). However, at the end of the accelerated storage period (day 16), the high concentration of fucoxanthin was detected in different groups of the gummies. The fucoxanthin contents of each group at day 16 of accelerated storage were 2.96 ± 0.09 mg/L, 7.76 ± 0.19 mg/L, 11.95 ± 0.03 mg/L, and 26.24 ± 0.13 mg/L, respectively, which indicates good bioavailability of fucoxanthin in the gummy products during storage.

κ-carrageenan is a high molecular weight sulfated polysaccharide extracted from seaweed, which has edible safety and antioxidant activity. Studies have shown that the antioxidant activity of carrageenan is proportional to the content of sulfate ester (Rocha de Souza et al., 2007). As for κ-carrageenan used in the gummies, the sulfated ester content is about 25%–30%, which is the highest among the edible carrageenan (Pourramezan et al., 2022). According to the study of (Arijón et al., 2023), UP could be used as a source of carrageenan, and the sodium alginate content in UP could improve the stability of gel structure. Therefore, carrageenan and UPP added to the gummies improved the stability of fucoxanthin.

#### 3.7.2 Antioxidant changes of seaweed gummy

Depicted in Fig. 3B is the antioxidant effect of the four types of seaweed gummies during accelerated storage. Without the addition of fucoxanthin in the gummy, the sample showed lower DPPH scavenging rate, which was <15%. The results revealed that some bioactive compounds in UPP may cause the antioxidative activity of seaweed gummies (Silva et al., 2022). As expected, DPPH scavenging rate promoted with the increase of fucoxanthin concentration, indicating that the addition of fucoxanthin exerted better antioxidant effect of the seaweed gummy. At the beginning of storage, DPPH scavenging rate of 0.5–1% fucoxanthin enriched seaweed gummies were 55.00 ± 0.99%, 60.08 ± 0.86%, and 70.66 ± 1.27%, respectively, which were the highest during the storage period. In the following period of the accelerated storage, the significant decrease of DPPH scavenging rate can be observed in Fig. 3B. It's worth noting that at the end of accelerated storage, 1% fucoxanthin gummy samples still showed a high DPPH scavenging rate, which was above 40%. The results were similar to the changes of fucoxanthin content in the gummies (Fig. 3A), which demonstrated the favourable stability of fucoxanthin in gummy products. At the end of the accelerated storage, DPPH scavenging rate of 0–1% fucoxanthin enriched seaweed gummies were 4.54 ± 2.03%, 18.24 ± 2.48%, 29.70 ± 2.06%, and 43.76 ± 0.72%, respectively.

#### 3.7.3 Texture change of seaweed gummies during accelerated storage

**Fig. 4:**
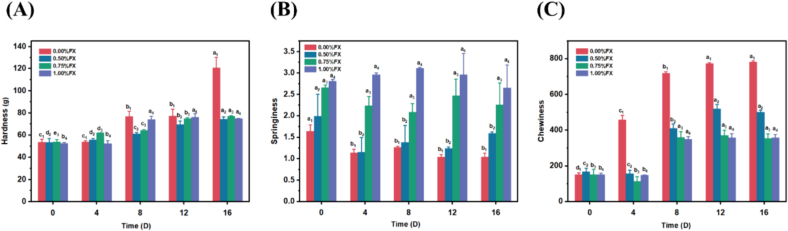
Texture changes of fucoxanthin seaweed eye-care gummies during accelerated storage. (A) Hardness. (B) Springiness. (C) Chewiness. Different letters with the same subscript number indicate significant difference (*P* < 0.05).

Hardness and springiness changes of the seaweed gummies during accelerated storage are the essential indicators of the texture quality. With the extension of storage time, the hardness of all groups gradually increased (Fig. 4A), which is due to the water loss during accelerated storage (Ding et al., 2018). As shown in Fig. 4B, the long-term high temperature and humidity of storage resulted in a decrease of springiness of the gummies. The results suggested that the growth of microbes during accelerated storage may damage the internal structure of the gummies which affected texture properties (Burgos-Díaz et al., 2022). However, compared with the gummy samples without fucoxanthin, the addition of fucoxanthin could maintain better springiness of the products in the accelerated storage period. Furthermore, the large number of hydroxyls contained in fucoxanthin could form hydrogen bonds with the hydroxyls of κ-carrageenan in the gummies. This conjugation could form a compact spatial network structure in the gummies, thereby enhancing the springiness (Q. Zhang et al., 2008). Moreover, the higher gel strength created denser networks in the gummy, which improved the springiness by trapping large volumes of water in the gummy (Z. Yu et al., 2022).

Hardness also determines the change in product chewiness. The chewiness of the four groups of gummies increased in a time-dependent manner, which was consistent with the trend of hardness changes during the storage (Fig. 4C). At day 16 of the storage, the gummies without fucoxanthin addition had the highest hardness and chewiness, and there were significant differences between the four groups. Fucoxanthin markedly maintained the hardness and chewiness of the gummies, which revealed a slight downtrend during the accelerated storage for 8–16 days (Fig. 4C). The results indicate that the addition of fucoxanthin could enhance the internal structural stability of the gummies, thus maintaining a better texture.

#### 3.7.4 Colour changes during accelerated storage of gummy candy

Table 1 shows the results of the colour changes of the gummies during accelerated storage. We found that at the beginning of storage (day 0), L* value of each group was relatively high. It's worth noting that the group without fucoxanthin addition showed the brightest colour. L* value revealed a decreasing trend with the increasing concentration of fucoxanthin in the gummies. As the accelerated storage period was prolonged, L* value of the gummies decreased. However, when a higher concentration fucoxanthin was used, the seaweed gummies presented better luster after 16 days of accelerated storage, increasing the L* value by 40% compared to the group without fucoxanthin addition (Table 1).

**Table 1:** The change of colour of FX seaweed eye-care soft sweets during storage.

	Time (D)	L*	a*	b*
0.00% FX	0	39.62 ± 1.60^Aa^	−2.51 ± 0.10^Cc^	14.10 ± 0.86^Aa^
	4	30.32 ± 0.45^Bb^	−1.30 ± 0.04^Cb^	4.95 ± 0.27^Bb^
	8	30.39 ± 0.33^Cb^	0.24 ± 0.03^Ca^	3.43 ± 0.19^Bc^
	12	27.33 ± 0.42^Cc^	0.26 ± 0.02^Ca^	2.50 ± 0.41^Bd^
	16	20.52 ± 0.44^Cd^	0.27 ± 0.04^Ca^	2.93 ± 0.11^Ccd^
0.50% FX	0	39.18 ± 2.77^Aa^	−2.10 ± 0.06^Be^	13.64 ± 0.53^Aa^
	4	31.21 ± 0.08^ABb^	−1.45 ± 0.11^Dd^	5.53 ± 0.23^Ab^
	8	31.28 ± 0.33^Bb^	0.50 ± 0.03^Ac^	5.39 ± 0.37^Ab^
	12	30.80 ± 0.48^Abc^	0.77 ± 0.05^Ab^	5.18 ± 0.18^Abc^
	16	28.75 ± 0.13^Ad^	1.00 ± 0.02^Aa^	4.59 ± 0.50^Bd^
0.75% FX	0	39.41 ± 1.15^Aa^	−1.94 ± 0.30^Bd^	13.70 ± 0.33^Aa^
	4	31.10 ± 1.05^ABb^	−1.14 ± 0.10^Bc^	4.91 ± 0.12^Bb^
	8	31.52 ± 0.32^Bb^	0.45 ± 0.05^Ab^	3.26 ± 0.32^Be^
	12	31.19 ± 0.31^Ab^	0.57 ± 0.02^Bab^	5.46 ± 0.09^Ac^
	16	28.57 ± 0.37^Ac^	0.80 ± 0.08^Ba^	6.24 ± 0.29^Ab^
1.00% FX	0	37.60 ± 0.32^Aa^	−1.34 ± 0.06^Ae^	13.63 ± 0.21^Aa^
	4	31.54 ± 0.32^Ac^	−0.28 ± 0.01^Ad^	3.93 ± 0.10^Cd^
	8	32.32 ± 0.44^Ab^	0.34 ± 0.02^Bc^	3.36 ± 0.22^Be^
	12	28.25 ± 0.08^Bd^	0.64 ± 0.05^Bb^	5.45 ± 0.18^Ac^
	16	26.42 ± 0.59^Be^	1.07 ± 0.07^Aa^	5.95 ± 0.31^Ab^

Data was shown as the means ± SDs. The statistical significance of differences was evaluated by one-way ANOVA followed by Duncan tests. Different letters indicate significant difference (*p* < 0.05).

Superscripts with different lower-case letters at different storage time indicate significant differences (*p* < 0.05). Superscripts with different capital letters at different concentrations indicate significant differences (*p* < 0.05).

The changes in the a* and b* values indicated that at the beginning of storage, the greenness of the four groups of gummies showed no significant difference (*P* > 0.05). We can observe in Table 1 that the greenness decreased as the accelerated storage time was prolonged. Additionally, with the increase of fucoxanthin concentration in the gummies, the greenness was gradually down regulated, suggesting that the reddish-brown colour of fucoxanthin mainly influenced a* and b* value during the accelerated storage (Sellimi et al., 2017). As the storage time was prolonged, the yellowness of the fucoxanthin enriched gummies were higher than none-fucoxanthin group. These results were also consistent with the above results that fucoxanthin contributed greatly to the b* value, and the UPP content in the gummies was easy to browning under the accelerated test condition during the storage. Taken together these results suggest that the addition of fucoxanthin showed a remarkable change to the colour of the seaweed gummies, leading to an increase in the yellowness and a decrease in the greenness of the gummies. The results were also like the study of (Sellimi et al., 2017).

### 3.8 Effect of other ingredients in gummies on the stability of fucoxanthin during *in vitro* digestion

The results of fucoxanthin changes in the gummy indicated that UPP and κ-carrageenan components in gummies protected the stability of fucoxanthin during accelerated storage. Therefore, the simulated *in vitro* digestion method was used to investigate the effect of the main components on the stability of fucoxanthin.

**Fig. 5:**
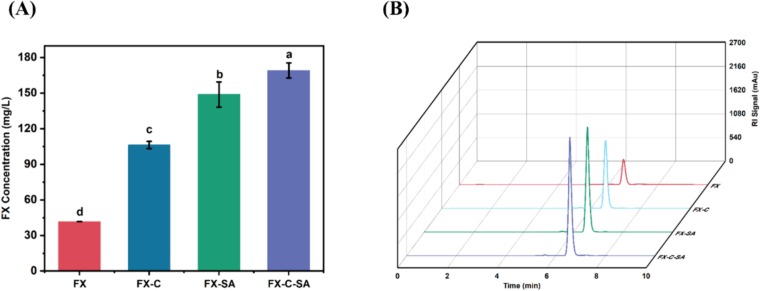
Effect of the main components in gummies on the retention rate of fucoxanthin during *in vitro* digestion. (A) HPLC chromatogram of fucoxanthin. (B) Concentration of fucoxanthin. FX, fucoxanthin; FX-C, fucoxanthin and κ-carrageenan; FX-SA, fucoxanthin and sodium alginate; FX-C-SA, fucoxanthin, κ-carrageenan and sodium alginate. Different letters indicate significant difference (*P* < 0.05).

Fig. 5 illustrates the bioavailability of fucoxanthin during *in vitro* digestion. According to the HPLC chromatogram, the retention time of fucoxanthin was about 6.5 min. The concentration of FX group after *in vitro* digestion was 41.65 ± 0.07 mg/L (Fig. 5A). Notably, the concentration of fucoxanthin showed a significant increase after the digestion with sodium alginate and κ-carrageenan. After the digestion with equal amount of κ-carrageenan and sodium alginate, the fucoxanthin content in the digestion samples increased to 106.32 ± 3.04 mg/L and 148.83 ± 10.59 mg/L, respectively. The retention rate of fucoxanthin reached the highest to 169.12 ± 6.33 mg/L in FSC group after the *in vitro* digestion, which was about four times higher than FX group. Results showed the protective effects of other contents in the gummy, which was the same as data revealed in the accelerated storage (Fig. 3). For carrageenan, only about 9% of carrageenan is excreted slowly in the urine, suggesting that carrageenan is absorbed through the small intestine and distributed to the organs through the circulation (Wang et al., 2024). It has been shown that sodium alginate can tolerate the environmental conditions of the upper gastrointestinal tract, but is destroyed by intestinal microorganisms to release the active substance (C. Zhang et al., 2022). This demonstrates the protective effects of κ-carrageenan and sodium alginate on fucoxanthin, which can prevent the degradation in the gastrointestinal tract. Moreover, the study indicated that the hydrogel beads obtained by the mixture of sodium alginate and κ-carrageenan were significantly effective in inhibiting the release of active ingredients from SGF owing to the presence of sulfated groups, compared to sodium alginate hydrogel beads (Sarıyer et al., 2020). Furthermore, the mixed digestion of the three compounds may cause the combination of different chemical bonds during *in vitro* digestion, which increased the bioavailability of fucoxanthin (Yuan et al., 2023). Therefore, we further studied the composition of the digestion products of different groups.

### 3.9 Identification of the *in vitro* digestion products in different groups

The MS identification results of the digestion products of FX, FS, FC, and FSC groups are shown in the **supplementary data**. After the *in vitro* digestion, fucoxanthin could be found in the four groups. The digestion products were identified according to our previous study (data not shown). In the FS group, a total of 10 digestion products were identified, with molecular weights distributed between 227 and 637. Compared with the digestion products of fucoxanthin, 5 new products included MINEs-445,040, UNPD218585, 3-(4-hydroxy-3-methoxyphenyl) propyl-2-propenoic acid, UNPD120475, and ester terpenoids were found. It can be observed that the β-mannuronic acid and guluronic acid structures of sodium alginate were found in some of the ester terpenoid compounds identified in the FS group, indicating the combination reaction during the simulated digestion process.

After the *in vitro* digestion with carrageenan (FC group), a total of 10 compounds were identified in common with FX group, and the distribution range of digestion product molecular weights was the same as FS group. The digestion products also included diterpenoids UNPD50430 and flavonoids, which may have been reassembled from furanohexose residues degraded by κ-carrageenan during *in vitro* digestion (Tecson et al., 2022), and combined with unstable propadiene bonds in fucoxanthin. Notably, flavonoids contributed to regulating gut microbiota (Li, Ren, et al., 2023). As shown in FSC group, 7 compounds were identified according to the MS data, with the molecular weights ranging from 287 to 637. Compared with the results of FX group, FC and FS groups, the proportion of low molecular weight products is relatively reduced in FSC group. The results indicated that sodium alginate and κ-carrageenan may prevent the degradation of fucoxanthin into small molecule compounds during *in vitro* digestion. Moreover, flavonoids formed by the combination of carrageenan degradation products with fucoxanthin have the potential to the regulation of gut microbiota.

## 4 Conclusion

Fucoxanthin enriched seaweed eye-care gummies could improve the cell viability of UVB-induced RMCs, which revealed potential photoprotection effect. Further validation of the bioactivity of the gummy can also be achieved through *in vivo* experiments. The addition of fucoxanthin ensured better texture and sensory properties of the gummy product. The gummy product showed significant antioxidant effects, among which 0.5% fucoxanthin group had the best sensory acceptability. During the accelerated storage period, fucoxanthin improved the springiness and chewiness of the seaweed gummy. At the end of the accelerated storage period, the fucoxanthin enriched gummies still maintained antioxidant effect, indicating that the product had a high stability during storage.

The other components sodium alginate and carrageenan in the gummies could improve the bioavailability of fucoxanthin. According to the MS data of the digested products, the low molecular weight digestion products were not identified in FSC group, indicating that the main components in the gummy improved the stability of fucoxanthin during simulated digestion process *in vitro*. In summary, the fucoxanthin seaweed eye-care gummies prepared in this study have potential photoprotective effects, which provides a theoretical basis for the development of fucoxanthin eye-care functional foods.

## CRediT authorship contribution statement

**Yu Liu:** Writing – review & editing, Methodology, Investigation, Formal analysis, Data curation. **Yixin Shi:** Writing – original draft, Formal analysis, Data curation, Conceptualization. **Yuting Wang:** Methodology, Formal analysis, Data curation. **Zhipeng Wang:** Methodology, Formal analysis. **Yuze Wang:** Methodology, Data curation. **Yujing Lu:** Methodology, Formal analysis, Data curation. **Hang Qi:** Writing – review & editing, Supervision, Resources, Investigation, Funding acquisition, Formal analysis, Conceptualization.

## Declaration of competing interest

The authors declare that they have no known competing financial interests or personal relationships that could have appeared to influence the work reported in this paper.

## Data Availability

The authors do not have permission to share data.

## Appendix A Supplementary data

Supplementary material 1 Image 1

Supplementary material 2 Image 2
