# Supplementary material for: A novel fucoxanthin enriched seaweed gummy: Physicochemical qualities and protective effect on UVB-induced retinal müller cells
Source: Food Chem X. 2024 Jul 14;23:101648. doi: 10.1016/j.fochx.2024.101648 (PMC11304860; doi:10.1016/j.fochx.2024.101648)
Supplement: Supplementary file 1 — Supplementary material 1 [file mmc1.docx]

Supplementary materials for

**A novel** **fucoxanthin enriched seaweed gummy: Physicochemical qualities and protective effect on UVB-induced** **retinal müller cells**

Yu Liu, Yixin Shi, Yuting Wang, Zhipeng Wang, Yuze Wang, Yujing Lu, Hang Qi*

National Engineering Research Center for Seafood, State Key Laboratory of Marine Food Processing and Safety Control, Collaborative Innovation Center of Provincial and Ministerial Co-construction for Seafood Deep Processing, Liaoning Province Collaborative Innovation Center for Marine Food Deep Processing, Dalian Technology Innovation Center for Chinese Pre-made Food, College of Food Science and Technology, Dalian Polytechnic University, Dalian 116034, China.

* Corresponding author: Hang Qi, Tel.: +86 411 86318785; Fax: +86 411 86323262, *E-mail address: qihang@dlpu.edu.cn

Table S1. Effects of different concentrations of FX on the texture of seaweed eye-care gummies

|  | Hardness (g) | Springiness | Chewiness |
| --- | --- | --- | --- |
| 0.00% FX | 51.92 ± 0.62^b^ | 1.31 ± 0.02^a^ | 121.39 ± 2.85^a^ |
| 0.50% FX | 52.43 ± 0.84^ab^ | 2.34 ± 0.03^a^ | 122.07 ± 5.92^a^ |
| 0.75% FX | 52.51 ± 0.23^ab^ | 2.17 ± 0.01^a^ | 122.55 ± 7.32^a^ |
| 1.00% FX | 51.07 ± 0.08^a^ | 2.29 ± 0.01^a^ | 123.48 ± 6.98^a^ |

Data was shown as the means ± SDs. The statistical significance of differences was evaluated by one-way ANOVA followed by Duncan tests. Different letters indicate significant difference (*P* <0 .05).

Table S2. Effects of different concentrations of FX on the color of seaweed eye-care gummies

|  | L* | a* | b* |
| --- | --- | --- | --- |
| 0.00% FX | 60.89 ± 0.49^a^ | -1.11 ± 0.33^a^ | 13.82 ± 0.04^a^ |
| 0.50% FX | 60.77 ± 0.09^a^ | -0.82 ± 0.13^a^ | 13.68± 0.11^a^ |
| 0.75% FX | 61.28 ± 0.39^a^ | -0.52 ± 0.03^b^ | 12.95 ± 0.14^a^ |
| 1.00% FX | 60.65 ± 0.06^a^ | -0.67 ± 0.05^b^ | 12.98 ± 0.09^a^ |

Data was shown as the means ± SDs. The statistical significance of differences was evaluated by one-way ANOVA followed by Duncan tests. Different letters indicate significant difference (*P* <0 .05).

Table S3. Sensory changes of FX seaweed eye-care gummies during storage

|  | Color | Appearance condition | Flavor | Taste | Overall |
| --- | --- | --- | --- | --- | --- |
| 0.00% FX | 18.65 ± 3.56^a^ | 18.05 ± 3.47^a^ | 14.70 ± 3.82^b^ | 15.40 ± 4.57^ab^ | 66.80 ± 8.48^a^ |
| 0.50% FX | 20.30 ± 2.70^a^ | 17.70 ± 2.64^a^ | 17.80 ± 3.43^a^ | 16.80 ± 4.05^a^ | 72.60± 8.52^a^ |
| 0.75% FX | 11.80 ± 4.85^b^ | 14.00 ± 4.05^b^ | 16.25 ± 3.96^ab^ | 16.75 ± 3.54^a^ | 58.80 ± 10.68^b^ |
| 1.00% FX | 9.15 ± 3.98^c^ | 11.50 ± 4.56^c^ | 17.05 ± 3.78^ab^ | 13.20 ± 6.78^b^ | 50.90 ± 10.73^c^ |

Data was shown as the means ± SDs. The statistical significance of differences was evaluated by one-way ANOVA followed by Duncan tests. Different letters indicate significant difference (*P* <0.05).


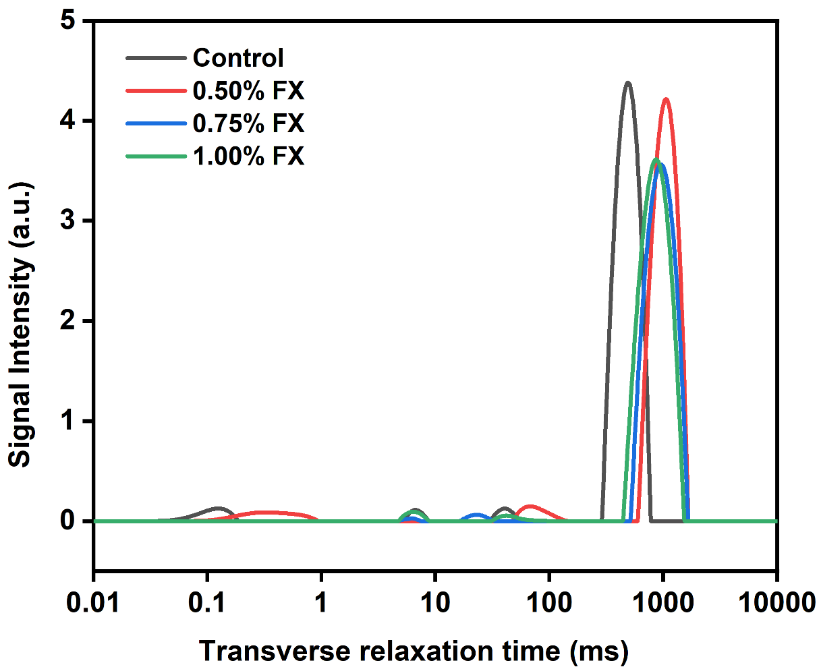


Figure S1. The relaxation spectrum of different concentrations fucoxanthin seaweed eye-care gummies.
